# Supplementary material for: Money attitudes, financial capabilities, and impulsiveness as predictors of wealth accumulation
Source: PLoS One. 2022 Nov 21;17(11):e0278047. doi: 10.1371/journal.pone.0278047 (PMC9678286; doi:10.1371/journal.pone.0278047)
Supplement: Attachment — Submitted filename: PlosOne Review.pdf [file pone.0278047.s001.pdf]

Money attitudes, financial capabilities, and impulsiveness as predictors of wealth accumulation

This paper examined the association between money attitudes, financial capabilities, impulsiveness, and wealth accumulation using a large sample of U.K. based respondents. The results revealed that demographic differences accounted for 27%, money attitudes accounted for 14%, and financial capability accounted for 24% of the variances in wealth accumulation. The authors also found that planning ahead, age, and income were consistently significant predictors of wealth.

Although, the findings from this study are rather incremental than novel, the study confirms the findings from past literature (that were mostly conducted with small primary datasets), by empirically testing the hypotheses, using a large-scale dataset. I think the value of this research and this important contribution of the study has been undersold, and the authors need to highlight this significant contribution of their research.

Comments and Suggestions

Analysis

Considering the heterogeneity of the readership of this journal, mentioning the purpose of reporting Hotelling's Trace (Hotelling-Lawley) in the regression analysis will be informative to the readers.

Low Chronbach's alpha for 'Choosing products' and 'staying informed'. Some justification on why it maybe fine to use 'choosing products', 'staying informed', and the money attitude variables, inspite of their low alpha values will be useful.

Page 20

"We did not find support in the regression analysis for our third hypothesis, about buying impulsiveness, though all the correlations were negative as hypothesised. Impulsivity in all spheres is associated with immaturity, poor emotional regulation and negative social outcomes [41]. It may be that effects of buying impulsivity on wealth are indirect for example accounted for via planning ahead and choosing products with which it correlates".

But in Table 3, 'buying impulsiveness' has a positive beta 0.01 ($p<0.01$) for savings and other financial investments, and a beta of 0.02 ($p<0.01$) for value of physical items. Doesn't this indicate that the negative association (as hypothesized) between impulsiveness and wealth accumulation goes away when other control variables are included in the model? Some clarification from the authors will be useful here.

Contribution of this study

Although the findings from this study are consistent based on the expectations and findings drawn from prior literature, and the contribution of this study appears incremental rather than novel; the results run with an incredibly rich dataset of over 90,000 respondents does provide

validity to the findings from previous studies. I believe this is an important contribution of this study.

Additional Limitations and Future Directions

Potential Endogeneity

Inspite of the rich data that has been used, it is still cross-sectional, and therefore the causality between variables, for example, ‘making ends meet’ and ‘saving and other financial investments’ may not be completely determined. This is because financial hardship or low savings might be driving the inability to make ends meet rather than not being able to make ends meet predicting lower levels of savings and other financial investments. This type of causality can be further analyzed using panel data analysis. I suggest that the authors include the use of cross-sectional dataset (instead of panel data) as a limitation of their study.

Possibility of Geospatial Analysis

The authors mention that geocodes were available for this dataset? If this is the case, then perhaps in future more research can be done by controlling for neighborhood/regional characteristics using the geospatial correlates. Although, this was not the purpose of this study, I think it will be beneficial to inform the readers of this possibility for those who maybe interested in pursuing this line of research in the future.

Potential for Generalizability

Although, the study uses a large dataset, it is a data of U.K. respondents. But, there is potential to replicate this study in other parts of the world, and this can be suggested in the future directions section of this manuscript.

Other Minor Points

Page 12: Results

“The correlations between all independent variables and the three dependent (wealth) variables are significant at the $p< .5$ level with most significant at $p<.001$”.

I believe this is a typographical error, and the authors really mean $p<0.05$ instead?

The paragraph indent on page 20, and a few other places do not seem correct. Overall, another round of copy editing will benefit this manuscript.
